# Comparison of deep learning with regression analysis in creating predictive models for SARS-CoV-2 outcomes

**DOI:** 10.1186/s12911-020-01316-6

**Published:** 2020-11-19

**Authors:** Ahmed Abdulaal, Aatish Patel, Esmita Charani, Sarah Denny, Saleh A. Alqahtani, Gary W. Davies, Nabeela Mughal, Luke S. P. Moore

**Affiliations:** 1 Chelsea and Westminster NHS Foundation Trust, 369 Fulham Road, London, SW10 9NH UK; 2 National Institute for Health Research Health Protection Research Unit in Healthcare Associated Infections and Antimicrobial Resistance, Imperial College London, Hammersmith Campus, Du Cane Road, London, W12 0NN UK; 3 King Faisal Specialist Hospital and Research Centre, Riyadh, Saudi Arabia; 4 Johns Hopkins University, Baltimore, MD USA; 5 North West London Pathology, Imperial College Healthcare NHS Trust, Fulham Palace Road, London, W6 8RF UK

**Keywords:** COVID-19, Coronavirus, Machine learning, Artificial intelligence, Prognostication

## Abstract

**Background:**

Accurately predicting patient outcomes in Severe acute respiratory syndrome coronavirus 2 (SARS-CoV-2) could aid patient management and allocation of healthcare resources. There are a variety of methods which can be used to develop prognostic models, ranging from logistic regression and survival analysis to more complex machine learning algorithms and deep learning. Despite several models having been created for SARS-CoV-2, most of these have been found to be highly susceptible to bias. We aimed to develop and compare two separate predictive models for death during admission with SARS-CoV-2.

**Method:**

Between March 1 and April 24, 2020, 398 patients were identified with laboratory confirmed SARS-CoV-2 in a London teaching hospital. Data from electronic health records were extracted and used to create two predictive models using: (1) a Cox regression model and (2) an artificial neural network (ANN). Model performance profiles were assessed by validation, discrimination, and calibration.

**Results:**

Both the Cox regression and ANN models achieved high accuracy (83.8%, 95% confidence interval (CI) 73.8–91.1 and 90.0%, 95% CI 81.2–95.6, respectively). The area under the receiver operator curve (AUROC) for the ANN (92.6%, 95% CI 91.1–94.1) was significantly greater than that of the Cox regression model (86.9%, 95% CI 85.7–88.2), *p* = 0.0136. Both models achieved acceptable calibration with Brier scores of 0.13 and 0.11 for the Cox model and ANN, respectively.

**Conclusion:**

We demonstrate an ANN which is non-inferior to a Cox regression model but with potential for further development such that it can learn as new data becomes available. Deep learning techniques are particularly suited to complex datasets with non-linear solutions, which make them appropriate for use in conditions with a paucity of prior knowledge. Accurate prognostic models for SARS-CoV-2 can provide benefits at the patient, departmental and organisational level.

## Background

Severe acute respiratory syndrome coronavirus 2 (SARS-CoV-2) has led to healthcare crises in several countries and remains disruptive in several others [1]. Accurately predicting patient outcomes would aid clinical staff in allocating limited healthcare resources and establishing appropriate ceilings of care, thereby mitigating the pressure on hospital departments. It would also allow service managers and policy makers to respond efficiently to possible future surges of SARS-CoV-2, the magnitudes of which may otherwise be difficult to predict [2].

There are a variety of methods which can be used to develop prognostic models, ranging from logistic regression and survival analysis to more complex machine learning algorithms and deep learning [3]. As a consequence of the emergent interest in deep learning, a number of techniques have been developed within this field with respect to the diagnosis, treatment and prognosis of the COVID-19 disease, including densely connected neural networks, recurrent networks and generative adversarial networks [4]. There is currently no consensus as to which of these techniques yields the most robust prognostic models [5], and whilst several models have been developed at a time when they are urgently required, there are a number of limitations which have impeded their use [6]. Several of the current models have been found to be highly susceptible to bias. For example, many demonstrate sampling bias as they excluded patients with no outcome at the end of the study period, leading to reported mortality rates of between 8 and 59% [6–9]. Others attempt to predict outcomes based on cross-sectional data, suggesting the outcome prediction is based on data which is likely collected at a different time to that for which the model is intended [10]. One model attempts to predict outcomes from the last measurements available in healthcare records [8]. Other limitations include the use of subjective predictors [6], small patient numbers [11], and considering suspected and confirmed SARS-CoV-2 cases as one group [12].

We aimed to develop and compare two separate predictive models using regression analysis and an artificial neural network (ANN) using the Transparent Reporting of a multivariable prediction model for Individual Prognosis or Diagnosis (TRIPOD) guidelines [13]. The models aim to predict the risk of death during admission in patients with SARS-CoV-2. We then compare the two techniques to establish whether deep learning could supplant classical methods in the context of an evolving pandemic.

## Methods

### Participants

All admitted patients with a laboratory diagnosis of SARS-CoV-2 during March 1–April 24, 2020 (i.e. a high prevalence period) from a single west London hospital were identified. Patients were included if they were admitted to hospital and diagnosed with SARS-CoV-2 based on real-time reverse transcriptase polymerase chain reaction (RT-PCR, proprietary Public Health England Assay until 10 March 2020, then AusDiagnostics^®^, Australia, assay thereafter). No patients were excluded.

Inpatients had their symptoms and clinical course documented in their electronic healthcare record (EHR) by the admitting clinical team (Millennium: Cerner Corporation, Kansas City, Missouri). Demographic and clinical data were extracted retrospectively from the EHRs for all patients included in the analysis by the infectious diseases team. Patient outcomes were followed up until death or discharge.

### Outcome measure

Outcome was defined as death occurring during hospital admission for patients who were admitted with a laboratory confirmed diagnosis of SAR-CoV-2.

### Predictors

Predictors were chosen in concordance with previously published literature [10, 14, 15], and included demographic details (age and sex), comorbidities (chronic respiratory disease, obesity, hypertension, diabetes, ischaemic heart disease, cardiac failure, chronic liver disease, chronic kidney disease, and history of a cerebrovascular event), symptomatology (fever, cough, dyspnoea, myalgia, abdominal pain, diarrhoea and vomiting, confusion, collapse, and olfactory change), and the number of days of symptoms prior to admission. Length of hospital stay to discharge, or death, was recorded for all patients to allow for survival analysis in the Cox regression model. Smoking history and ethnicity data were not included in the predictive models due to 28.9% and 23.4% of patients having missing data for these fields, respectively.

Age and number of days of symptoms prior to admission were continuous variables. All other predictors were encoded as binary presence features. Sex was converted to a binary feature where 0 and 1 represented male and female patients, respectively. Predictors were chosen such that they can be elicited on first contact with a healthcare worker. The intended use, for both models, is therefore an outcome prediction based on clinical admission data.

### Statistical analysis

Patient baseline characteristics were described by mean and median for continuous variables and frequency and proportion for categorical variables. Log rank analysis was applied to the whole dataset to report unadjusted associations between each predictor and the outcome. Age was not normally distributed and was normalised by calculating its fractional ranks and then using an inverse density function. We then used an independent samples t-test to compare age by outcomes. Number of days of symptoms prior to hospital admission (NOD) were also not normally distributed and a Mann–Whitney U test was carried out to compare NOD between outcome groups. Multivariable Cox regression analysis was then applied to contextualise the predictors in relation to each other.

### Cox regression predictive model

To create a predictive model for death in SARS-CoV-2, we randomly split the dataset into training (80%) and test (20%) sets. As others have demonstrated, the optimal proportion of the dataset partitioned for training depends on the full dataset size and classification accuracy, with higher accuracies and smaller dataset sizes requiring a larger majority of the data for training the model [16]. However, a range of proportions for the training set were trialled during the training phase of model development for both the Cox regression and ANN models. The training/test set portions yielding the highest average area under the receiver operator curve (AUROC) during training cross-validation were used in the testing phase, and their results are reported in this analysis. On the training set, we used a parsimonious model building approach using the clinically relevant demographic, comorbidity and symptomatology features identified. All predictors were included in a Cox regression model irrespective of whether they were significant in univariable log-rank analysis. Using k-fold cross-validation on the training set, we chose the model with the lowest Akaike information criterion (AIC) score and highest concordance index (c-index) [17]. Subsequently, predictors which were not significantly associated with death were removed using backwards elimination. This generated a list of predictors making up a predictive model. We then assessed the performance of the model by calculating the survival function at the third quartile of length of stay for patients in the test set, as length of stay was not normally distributed. Since predicting mortality is a binary classification problem, a standard threshold of 0.5 (50%) was used to predict mortality. For example, if the model predicts a patient-specific mortality of 60%, this is interpreted as a “positive prediction”, in that the patient is likely to die. Accuracy, sensitivity, specificity, positive predictive value, and negative predictive value were computed. Using k-fold cross-validation on the whole dataset allowed for a calculation of a mean c-index with 95% confidence intervals (CI). Model calibration was assessed graphically using a calibration curve and numerically with a Brier score, which represents the mean squared error for a probabilistic forecast, with a lower score representing more calibrated predictions [18].

### Artificial neural network predictive model

**Fig. 1:**
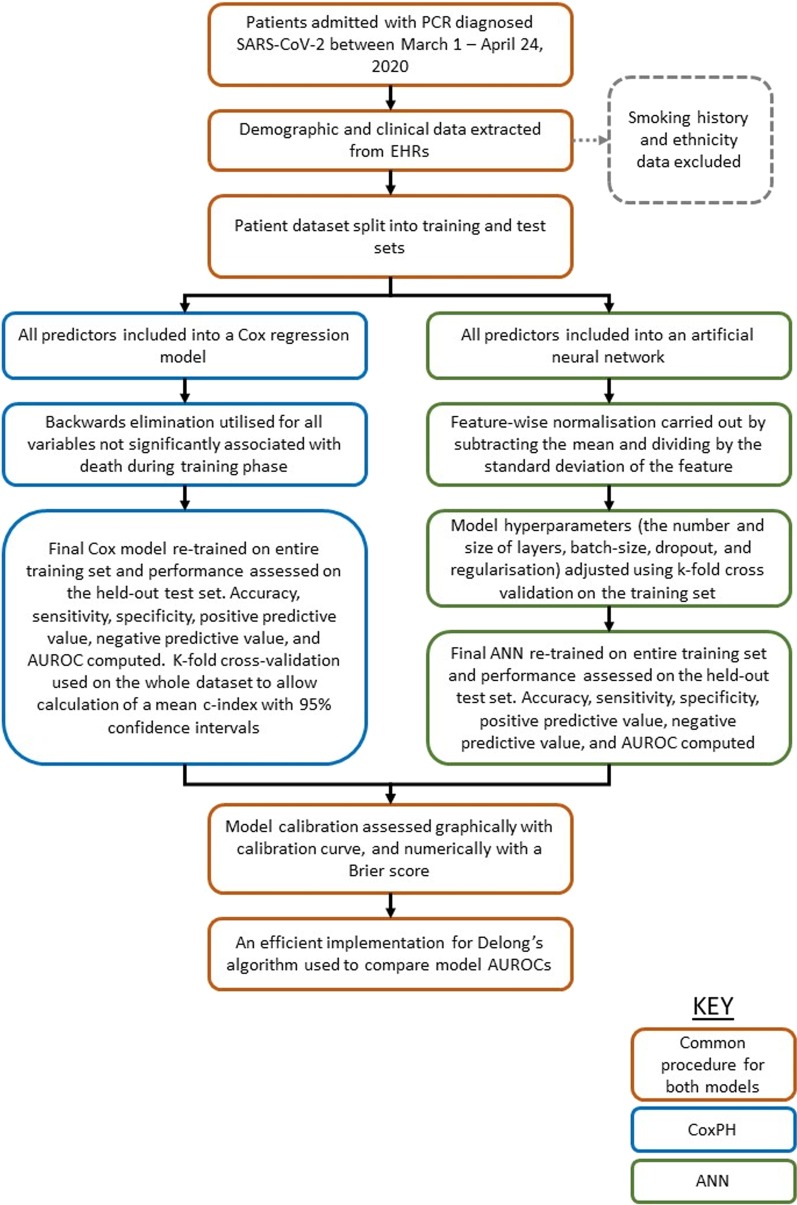
Summary of the methodology used in the development and assessment of two prognostic models for patients admitted with SARS-CoV-2 in a West London population, during March 1–April 23, 2020. *ANN* artificial neural network, *CoxPH* Cox regression model

The dataset was again randomly split into training (80%) and test (20%) sets. To maximise network learning efficiency, feature-wise normalisation was used. Each feature in the input data was centred around 0 by subtracting the mean of the feature, and then dividing it by its standard deviation [19]. The open-source TensorFlow machine learning library [20] was used to construct the ANN. To optimise the model, we adjusted hyperparameters (the number and size of layers, batch-size, dropout, and regularisation) using k-fold cross validation on the training set. The ANN was designed to achieve maximal performance on cross-validation. Once the model architecture was established, we retrained the ANN on the entire training set, before finally validating its performance on the test set. We calculated the same performance metrics and assessed calibration in the same manner as the Cox regression model. The performance profiles of the models were then compared, and an efficient implementation for Delong’s algorithm (which is an algorithm used to compare the area under two or more correlated receiver operator curves) was used to compare the AUROC between both models [21, 22]. Figure 1 illustrates a summary of the model development and assessment methodology.

## Results

### Participants

398 patients were identified, of which 95 died during hospital admission with SARS-CoV-2. 3/398 (0.75%) were still inpatients at the end of the follow-up period. There were no missing data in the variables used for analysis. Table 1 summarises the demographic, comorbidity, and symptomatology characteristics of the cohort, with the log rank (Mantel–Cox) analysis of all predictors. The mean age was 63.2 years and there was a statistically significant relationship between age and death (*p* < 0.001). The median number of days of symptoms prior to admission was 7 days (IQR 2–10). There was no significant association between number of days of symptoms and death (*p* = 0.09).

**Table 1:** Summary of demographics, comorbidities, symptoms, and outcomes of 398 patients admitted with SARS-CoV-2 in a West London population, during March 1–April 23, 2020

	Number (%)	Chi-Square	*p* value
*Demographics*			
Age (mean)	63.2 years		
Sex (M)	223 (56.0%)	3.54	
*Comorbidities*			
Cardiac failure	22 (5.5%)	79.56	*< 0.005*
Cerebrovascular event	29 (7.3%)	24.14	*< 0.005*
Chronic kidney disease	33 (8.3%)	49.25	*< 0.005*
Chronic liver disease	6 (1.5%)	5.915	*0.015*
Chronic lung disease	84 (21.1%)	6.01	*0.01*
Diabetes	104 (26.1%)	4.39	*0.036*
Hypertension	147 (36.9%)	17.88	*< 0.005*
Ischaemic heart disease	47 (11.8%)	37.63	*< 0.005*
Obesity	15 (3.8%)	0.015	0.903
*Symptoms*			
Abdominal pain	40 (10.1%)	2.6	0.11
Collapse	37 (9.3%)	55.5	*< 0.005*
Confusion	59 (14.8%)	117.35	*< 0.005*
Cough	247 62.1%)	3.05	0.081
Diarrhoea and vomiting	105 (26.4%)	4.64	*0.031*
Dyspnoea	223 (56.0%)	15.88	*< 0.005*
Fever	216 (54.3%)	0.12	0.73
Myalgia	68 (17.1)	0.002	0.97
Olfactory change	36 (9.0%)	1.11	0.29
Length of stay (median)	5 days		
Number of days of symptoms prior to admission (median)	7 days		
*Outcome*			
Death	95 (23.9%)		

The association of each predictor with death following log rank analysis (reported with the Chi-square statistic) is shown

### Cox regression model

#### Development

**Fig. 2:**
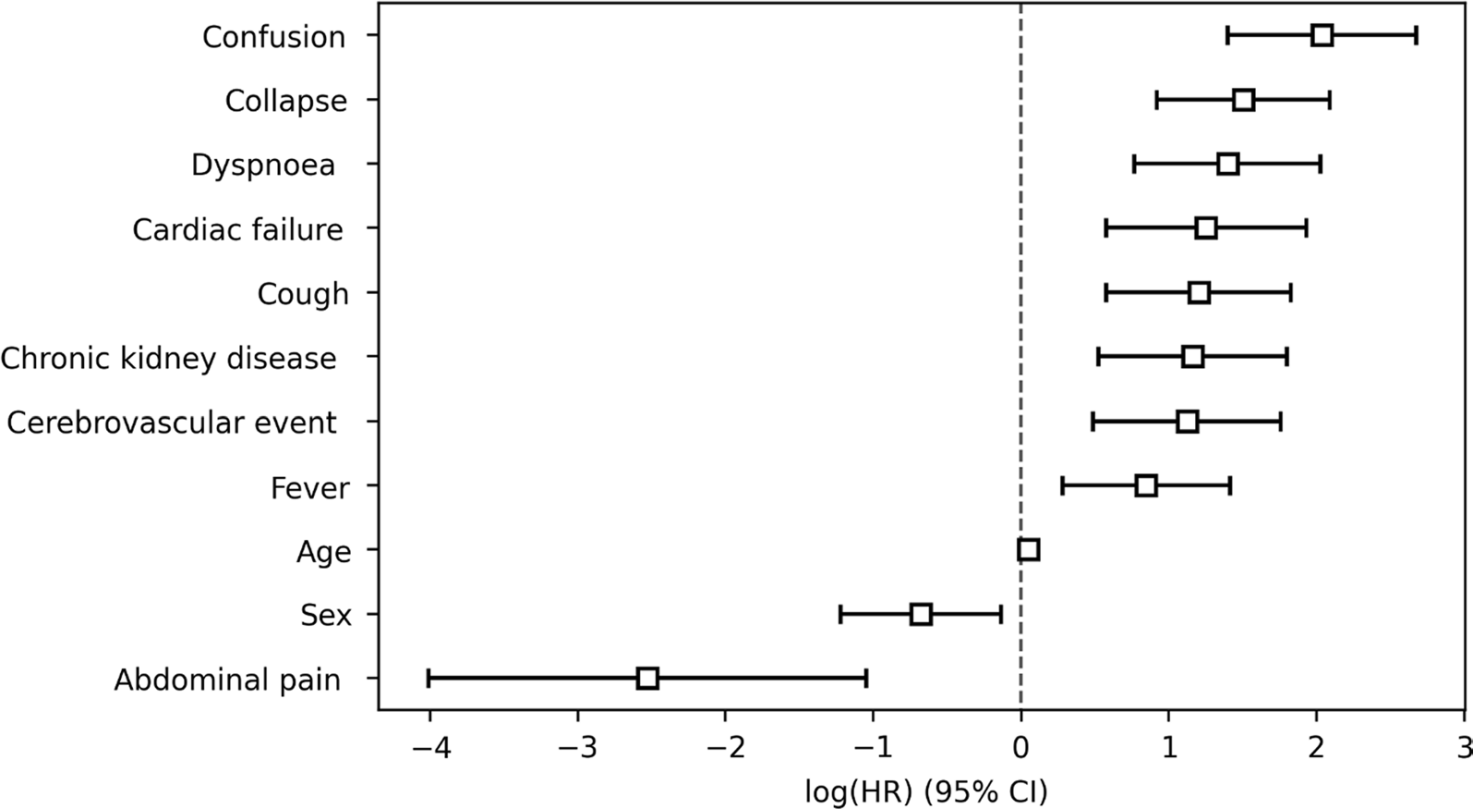
Cox prognostic model of demographics, comorbidities and symptoms, and the log hazard ratio of death in patients admitted with SARS-CoV-2 in a West London population, during March 1–April 23, 2020

Table 2 shows the association of all predictors with survival following multivariable analysis. Following backwards elimination on the training set (318/398), the remaining variables of significance were: age, sex, obesity, ischaemic heart disease, cardiac failure, chronic liver disease, chronic kidney disease, cerebrovascular event history, cough, dyspnoea, abdominal pain, confusion and collapse (Fig. 2). Goodness of fit testing showed an AIC = 572.93 with a c-index of 0.90 on the training set.

**Table 2:** Multivariable Cox regression analysis in 398 patients admitted with SARS-CoV-2 in a West London population, during March 1–April 23, 2020

Variable	Hazard ratio	Lower 95% CI	Upper 95% CI	*p* value
Age	1.05	1.03	1.07	*< 0.005*
Sex (F)	0.6	0.36	1	*0.05*
Cardiac failure	2.92	1.52	5.62	*< 0.005*
Cerebrovascular event	2.36	1.28	4.36	*0.01*
Chronic kidney disease	2.32	1.31	4.1	*< 0.005*
Chronic liver disease	3.52	1.2	10.35	*0.02*
Chronic respiratory disease	0.9	0.53	1.53	0.7
Diabetes	1.41	0.86	2.3	0.17
Hypertension	1.02	0.64	1.63	0.93
Ischaemic heart disease	2.09	1.29	3.4	*< 0.005*
Obesity	2.74	0.97	7.7	0.06
NOD	1.01	0.97	1.05	0.65
Abdominal pain	0.29	0.08	1.02	*0.05*
Collapse	4.21	2.4	7.41	*< 0.005*
Confusion	6.03	3.5	10.41	*< 0.005*
Cough	1.88	1.1	3.2	*0.02*
Diarrhoea/vomiting	1.32	0.68	2.54	0.41
Dyspnoea	3.49	1.93	6.32	*< 0.005*
Fever	1.88	1.13	3.13	*0.02*
Myalgia	1.43	0.69	2.94	0.33
Olfactory change	0.87	0.31	2.47	0.79

*NOD* Number of days of symptoms prior to hospital admission

#### Specification

We generated a model that calculates the hazard function determined by the following significant variables and their coefficients:

$$h\left(t\right)= {h}_{0}(t)\times \mathrm{exp}(\left(0.05\times age\right)+\left(-0.78\times sex\right)+\left(1.48\times obesity\right)+\left(0.93\times ischaemic heart disease\right)+\left(0.91\times cardiac failure\right)+\left(1.36\times chronic liver disease\right)+\left(1.03\times chronic kidney disease\right)+\left(0.78\times cerebrovascular event\right)+\left(1.01\times cough\right)+\left(1.54\times dypsnoea\right)+\left(-1.45\times abdominal pain\right)+\left(1.81\times confusion\right)+(1.35\times collapse))$$

Median length of stay was 7 days with the upper quartile being 13 days. Therefore, to predict outcomes, the survival function was calculated for patients at day 13 in the test set.

#### Performance

K-fold cross validated mean c-index on the training set was 89.0% (95% CI 84.2–94.3). When applied to the test set, the Cox regression model provided a sensitivity of 50.0% (95% CI 28.2–71.8), specificity of 96.6% (95% CI 88.1–99.6), positive predictive value of 84.6% (95% CI 57.0–95.8) and negative predictive value of 83.6% (95% CI 77.0–88.6) with an accuracy of 83.75% (95% CI 73.8–91.1). The c-index was 86.9% (95% CI 85.7–88.2). The final model had a Brier score of 0.13.

### ANN model

#### Development

**Fig. 3:**
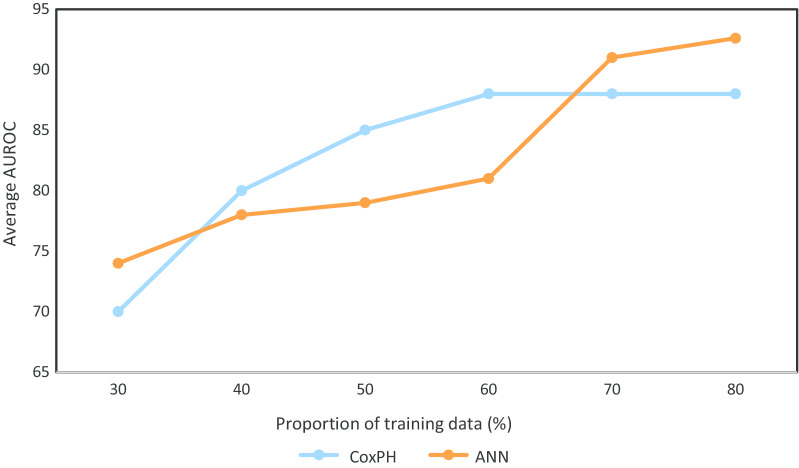
The average area under the receiver operator curve (AUROC) achieved by a Cox regression model and an Artificial Neural Network for a range of training set proportions in patients admitted with SARS-CoV-2 in a West London population, during March 1–April 23, 2020. *ANN* artificial neural network, *CoxPH* Cox regression model

We applied the ANN to the training set and adjusted the hyperparameters (layers, neurones, drop out, batch size, regularisation and epoch number) to achieve a model architecture providing the highest accuracy, AUROC and the lowest loss as measured by binary cross-entropy on the validation set. Once architecture was optimised, the model was retrained on the entire training set and evaluated on the test set. Figure 3 demonstrates the average AUROC by training proportion for both the Cox regression and ANN models during training cross-validation.

#### Specification

**Fig. 4:**
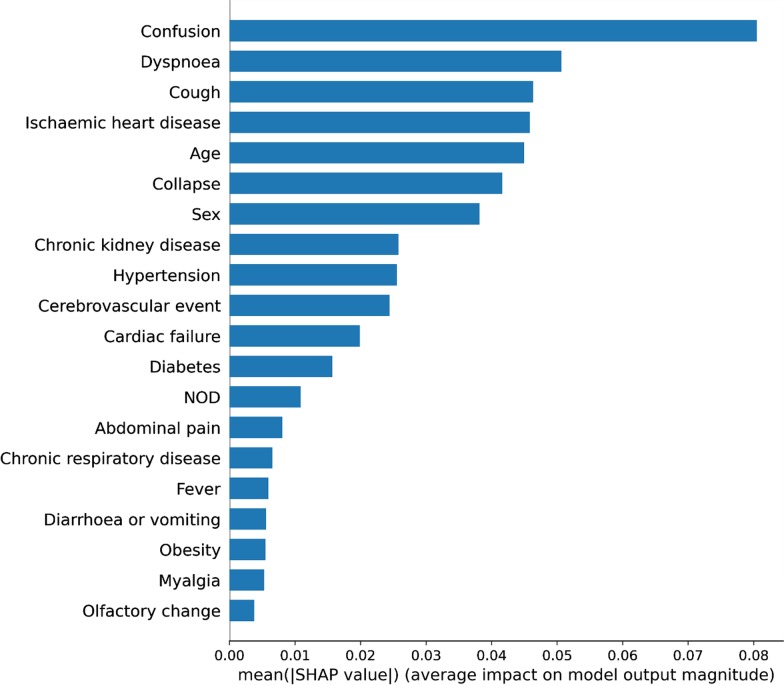
Predictor importance as considered by an Artificial Neural Network trained and validated on 398 patients with SARS-CoV-2 in a West London hospital, during March 1–April 24, 2020. *SHAP value* Shapley additive explanations value. This approximates how much each predictor contributes to the average prediction for the dataset. *NOD* number of days of symptoms prior to hospital admission

Optimal validation results were achieved with an ANN with 21 units in the input layer to match the input dimension of the dataset, that is, the total number of predictors. Two further hidden layers were used with 8 and 4 units, respectively. A dropout of 20% was applied to the input layer and both hidden layers to prevent overfitting. Regularisation did not substantively improve results and therefore no regularisation techniques were used. Figure 4 demonstrates the average impact on model output magnitude by each predictor.

#### Performance

**Fig. 5:**
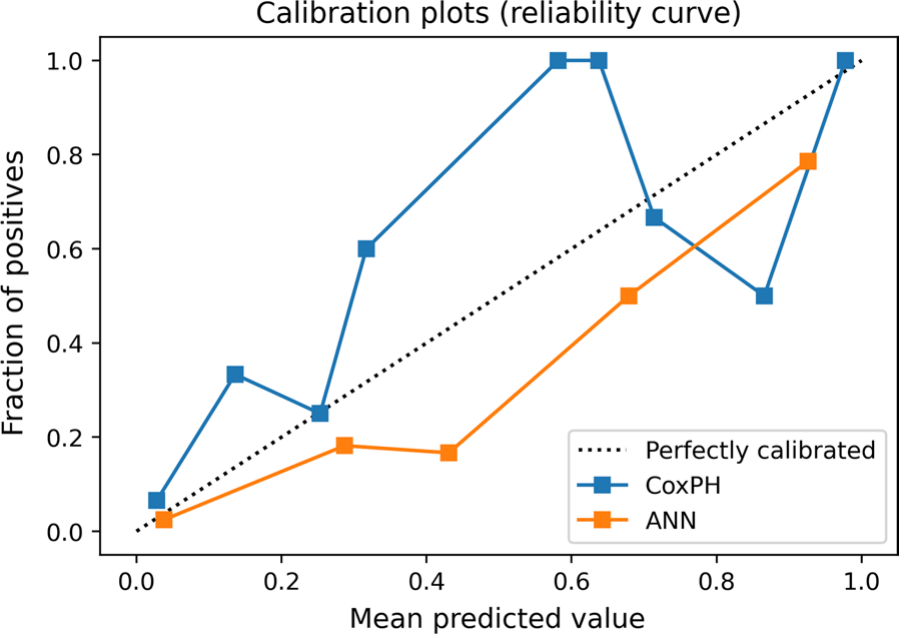
Calibration of a Cox regression model and ANN on 398 patients with SARS-CoV-2 in a West London hospital, during March 1–April 24, 2020. *ANN* artificial neural network, *CoxPH* Cox regression model

The final ANN architecture provided an accuracy of 88.1% and AUROC of 90.9% on cross validation. When applied to the test set, the model provided a sensitivity of 64.7% (95% CI 38.3–85.8), specificity of 96.8% (95% CI 89.0–99.6), positive predictive value of 84.6% (95% CI 57.4–95.7) and negative predictive value of 91.4% (95% CI 84.2–95.1), with an accuracy of 90.0% (95% CI 81.2–95.6). The AUROC on the test set was 92.6% (95% CI 91.1–94.1). The ANN had a Brier score of 0.11. Table 3 shows the performance metrics of each model. Figure 5 demonstrates the calibration of each model.

**Table 3:** Performance of the Cox regression model and an ANN on 398 patients with SARS-CoV-2 in a West London hospital, during March 1–April 24, 2020

	Cox regression model (95% CI)	ANN model (95% CI)	Cox regression vs ANN model
Sensitivity	50.0% (28.2–71.8)	64.7% (38.3–85.8)	
Specificity	96.6% (88.1–99.6)	96.8% (89–99.6)	
Positive predictive value	84.6% (57.0–95.8)	84.6% (57.4–95.7)	
Negative predictive value	83.6% (77.0–88.6)	91.4% (84.2–95.1)	
Accuracy	83.75% (73.8–91.1)	90.0% (81.2–95.6)	
AUROC	86.9% (85.7–88.2)	92.6% (91.1–94.1)	Z = 12.021, *p* < 0.001

*ANN* artificial neural network, *AUROC* area under the receiver operating characteristic curve

## Discussion

Two models were developed in accordance with TRIPOD methodology to predict death during hospital admission among SARS-CoV-2 patients. Both models demonstrate acceptable sensitivity and good specificity. Although both have good accuracy, the ANN has significantly greater discriminatory ability. Both models demonstrate acceptable calibration. Developing robust prognostic models for SARS-CoV-2 has benefits for the patient, medical departments, and hospital organisations.

Previous literature reports mixed performance of machine learning and deep learning techniques when compared to regression analysis [5]. Whilst machine learning does not obviate the need for classical methods [23], machine learning techniques have been shown to perform significantly better than classical regression models in high-dimensionality datasets [24]. Furthermore, ANNs have been shown to perform well on datasets of varying size [25–27]. Our results support the use of an ANN in a moderate sized, high-dimensional dataset, whilst having a non-inferior performance profile to a Cox regression model.

The Cox regression model used 11 predictors to calculate survival function, whilst the ANN uses all 21 input features, and attributes different weightings to each feature. Both models identify confusion, collapse, dyspnoea, cough, chronic kidney disease, heart failure, cerebrovascular event history, fever, and sex as more significant predictors of mortality. The ANN additionally identifies ischaemic heart disease and hypertension as important features. Abdominal pain is considered to have little effect on model output by the ANN, which is a significant ‘protective factor’ in the Cox model. In this context, abdominal pain may represent a milder form of SARS-CoV-2. These variables, in particular the comorbidities, have been shown to be associated with mortality in the current literature, such as the ISARIC protocol, which analysed 20,133 SARS-CoV-2 positive patients [28].

The Cox regression model accounts for censored patients in the study and therefore no patients were excluded on account of not having a recorded outcome at the end of the follow-up period. This avoids the introduction of sampling bias. The predictors chosen for inclusion in both models can be accrued from an initial encounter with a healthcare worker and relate to the underlying clinical condition of each patient. This has a dual benefit. Firstly, this standardises the data-collection process and ensures both models are compared on a congruent dataset. Secondly, the nature of the predictors means that the intended use of the models is clear in that they both produce a point-of-admission mortality prediction, which is particularly applicable to the development of medical calculators. The models analyse the outcomes for laboratory confirmed SARS-CoV-2 patients, eliminating potential bias introduced by including suspected cases who are subsequently diagnosed with other conditions.

The predictive models here do have several limitations, however. There are a variety of haematological and radiological predictors which have been associated with SARS-CoV-2 outcomes which are not included in our models [29, 30]. Whilst our current models can produce point-of-admission outcome predictions due to the relative ease of collecting demographic, comorbidity and symptom data, additional clinical parameters could be introduced in future to improve the predictive accuracy of the models. We could not account for patients who were admitted for, and diagnosed with SARS-CoV-2, but may have died due to another comorbidity. However, this likely represents a minority of patient deaths. The Cox regression model predicts survival function at day 13; whilst this accounts for the majority of hospital admission lengths, predicting survival in this way may overestimate survival chance for outliers who died at a later date. In contrast, the ANN model produces an overall risk prediction irrespective of length of admission. However, given the median length of stay of 7 days with an upper quartile of 13 days, predictions from the ANN should be used cautiously for longer lengths of hospital stay. There may be a delay between patient presentation and obtaining a laboratory diagnosis. Therefore, whilst it is possible to use either model at the point of admission, the prediction should only be applied to patients who have a confirmed diagnosis of SARS-CoV-2. Finally, data was collected at a single site during a period of high prevalence, and therefore results should be generalised with caution to other populations and those with a different SARS-CoV-2 prevalence.

A prospective, multi-centre analysis is required to further validate the model and improve generalisability of results. Machine learning techniques are ideal for fluctuating environments as they can adapt to new data. For example, using online/active learning, an ANN can train incrementally by being fed data instances sequentially. Each step is relatively fast and cheap, meaning the system can continuously learn as more data is available. This represents a major advantage relative to static statistical models [31]. Future research should focus on implementing adaptive workflows to allow for multi-site data collection, cross-population train/test modelling, and flexible systems which learn incrementally. Additionally, multimodal data (such as encoded radiographic data), and other potentially important parameters such as hospital capacity, testing capacity/rate and income versus commodities (poverty) can all be incorporated to produce more generalisable, highly-performant models [32, 33]. Furthermore, deep learning techniques such as recurrent neural networks can be used for time-series analysis, and therefore account for important events such as ICU admission as they occur. This may represent an additional avenue for further research. Finally, other decision points in SARS-CoV-2 patient journeys need to be predicted, and adapting the models to predict need for antibacterial agents for secondary infection [34], or for steroids where indicated [35], are clear avenues for exploration.

## Conclusion

Accurate prognostic models for SARS-CoV-2 can provide benefits at the patient, departmental and organisational level. Such models could optimise the response to possible future surges of SARS-CoV-2. We demonstrate an ANN which is non-inferior to a Cox regression model but has the potential for further development such that it can learn as new data becomes available. Deep learning techniques are particularly suited to complex datasets with non-linear solutions, which make them appropriate for use in conditions with a paucity of prior knowledge, such as in SARS-CoV-2 infection.

## Data Availability

The datasets analysed during the current study and further details on gaining access to the intervention reported within this study are available from the first author (AA ahmed.abdulaal@nhs.net) on reasonable request, as long as this meets local ethics and research governance criteria. The open source machine learning framework Tensorflow 2.1.0 (https://github.com/tensorflow/tensorflow) was used to develop the neural network. The architecture was written in the python programming language (Python 3.7.7). Scikit-learn 0.22 and its dependencies were utilised to create the data pre-processing pipeline and to create the graphs in this analysis.

## Abbreviations

AIC: Akaike information criterion
ANN: Artificial neural network
AUROC: Area under receiver operator curve
CI: Confidence interval
c-index: Concordance index
NOD: Number of days
SARS-CoV-2: Severe acute respiratory syndrome coronavirus 2
TRIPOD: The Transparent Reporting of a multivariable prediction model for Individual Prognosis or Diagnosis
UK: United Kingdom
